# Time for Change: A 3-Year Prospective Study on Mediterranean Diet Adherence and Body Composition in Kidney Transplant Recipients

**DOI:** 10.3390/healthcare13070840

**Published:** 2025-04-07

**Authors:** Marijana Vučković, Josipa Radić, Andrea Gelemanović, Andrej Belančić, Hana Đogaš, Mislav Radić

**Affiliations:** 1 Department of Internal Medicine, Division of Nephrology, Dialysis and Arterial Hypertension, University Hospital of Split, 21000 Split, Croatia; mavuckovic@kbsplit.hr (M.V.); josiparadic1973@gmail.com (J.R.); 2 Internal Medicine Department, School of Medicine, University of Split, 21000 Split, Croatia; 3 Mediterranean Institute for Life Sciences (MedILS), University of Split, 21000 Split, Croatia; agelemanovic@medils.unist.hr; 4 Department of Basic and Clinical Pharmacology and Toxicology, Faculty of Medicine, University of Rijeka, 51000 Rijeka, Croatia; andrej.belancic@uniri.hr; 5 Department of Neurology, University Hospital of Split, 21000 Split, Croatia; hana.dogas@gmail.com; 6 Department of Internal Medicine, Division of Rheumatology, Allergology and Clinical Immunology, University Hospital of Split, 21000 Split, Croatia

**Keywords:** Mediterranean diet, follow-up, nutritional status, kidney transplant

## Abstract

Background: The aim of this prospective follow-up study was to evaluate changes in body composition parameters and Mediterranean diet (MeDi) adherence among kidney transplant recipients (KTRs) over a three-year period. Additionally, this study sought to investigate the associations between these changes and clinical parameters, including laboratory parameters, new onset of diseases, and death outcome. Methods: A total of 116 KTRs were initially assessed in 2019 and subsequently re-evaluated in 2022. The Mediterranean Diet Serving Score (MDSS) was used to assess dietary adherence to the MeDi at baseline and follow-up assessments. Bioelectrical impedance analysis was used to assess body composition and clinical outcomes were assessed by the data available from medical charts. Results: After three years, MeDi adherence significantly decreased (*p* = 0.028) from 15 (18.29%) to 5 (6%), with dominantly lower adherence for vegetable, fruit, legume, red meat, olive oil and fish intake. Regarding body composition parameters, the most prominent change was seen in muscle mass, which deteriorated from 41.77% (IQR 6.46) to 39% (IQR 6.14; *p* = 0.004). However, changes in fat mass level were not significant in the follow-up period. Furthermore, cereal intake, fasting blood glucose (FBG), cholesterol level, level of low-density lipoprotein cholesterol (LDL), triglyceride leve and presence of diabetes mellitus lwere shown to be predictive for the decline of skeletal muscle mass. There were no significant changes in the estimated glomerular filtration rate (eGFR) or albuminuria level during the follow-up period. Associations with the death outcome were found for the MeDi-advised intake of eggs (β = −1.06, HR = 0.35, CI (0.14–0.87), *p* = 0.023), phase angle (PhA) (β = −2.68, HR = 0.07, CI (0.01–0.43), *p* = 0.004), cholesterol level (β = 0.95, HR = 2.60, CI (1.40–4.70), *p* = 0.001) and calcium level (β = −7.21, HR = 0.00, CI (0.00–1.50), *p* = 0.063). Conclusions: This study highlights a significant decline in MeDi adherence and skeletal muscle mass among KTRs over a three-year follow-up period, with no notable changes in fat mass or kidney function. The predictors of muscle mass loss and associations with mortality underscore the importance of dietary and clinical management in this population.

## 1. Introduction

The interplay of diet and body composition in kidney transplant recipients (KTRs) is an essential but overlooked field of research. Multiple studies have demonstrated the cardiovascular and metabolic advantages of the Mediterranean diet (MeDi), especially in patients with chronic kidney disease (CKD) and KTRs. It functions in controlling CKD consequences, including metabolic acidosis and hyperphosphatemia, while slowing the course of the disease [1]. Furthermore, by reducing glomerular hyperfiltration, a plant-based, low-protein diet like the MeDi may promote renal allograft health [2,3]. A significant finding in support of the MeDi promise for long-term renal outcomes is that Gomes-Neto et al. [4] showed that following the MeDi is linked to lower chances of kidney function decline and graft failure in KTRs.

KTRs are known to be at risk for both sarcopenia and obesity through multifactorial etiology, including corticosteroid-induced hyperphagia, cessation of dietary restrictions, lack of physical activity, psychological factors, and a lack of information about healthy eating patterns [5,6]. Parameters of nutritional status correlate to many clinical outcomes in this population. Obesity was associated with increased risk of death, delayed graft function, acute rejection, wound infection, dehiscence, and prolonged hospital stay in KTRs and is recommended to be assessed for all kidney transplant candidates using either the body mass index (BMI) or waist-to-hip ratio (WHtR) criteria [7]. Lean body mass was found to be associated with the left ventricular mass index and renal resistive index [8].

The parameters assessed by computed tomography pretransplant—the psoas muscle mass index and skeletal muscle index, which reflect muscle tissue, as well as sarcopenia—are predictors of 1-year transplant survival. In contrast, obesity and visceral adipose tissue, representing fat tissue, are shown as indicators of 3-year and 5-year transplant survival [9].

On the topic of importance of the long-term follow-up in this population speaks the study from Jiang et al. on 6116 KTRs. in Canada, which found significantly higher hospitalization rates compared to the general population, primarily due to infectious diseases and endocrine disorders. The hospitalizations peak was in the first-year post-transplant, with a decline over the next 2 to 3 years. After about 3 to 4 years, the rates stabilized. Overall, KTRs have been shown to have a relative risk of hospitalization approximately five times greater than that of the general population, underscoring their ongoing health challenges, especially in the early recovery period [10].

The evidence on changes of nutritional status and dietary habits, together with long-term follow-up data, is scarce in this population. Our previous cross-sectional study examined the association between MeDi adherence and nutritional status in Dalmatian KTRs. We found that 25% of our participants adhered to the MeDi, and the results showed some trends suggesting potential benefits of the MeDi [11]. These findings inspired us to undertake this prospective follow-up study. The aim of this prospective follow-up study was to evaluate changes in body composition parameters and Mediterranean diet (MeDi) adherence among KTRs over a three-year period. Additionally, the study sought to investigate the associations between these changes and clinical parameters, including laboratory parameters, new onset of diseases, and death outcome.

## 2. Materials and Methods

A sample of 116 KTRs who were 18 years or older, had functioning grafts, and with no mobility issues were included in this follow-up study. They were all examined in 2019 at the Department of Nephrology and Dialysis at the University Hospital of Split, Croatia.

The baseline examinations included clinical and laboratory data, anthropometric measurements, blood pressure measurement, body composition, and assessment of adherence to the MeDi [11].

**Figure 1:**
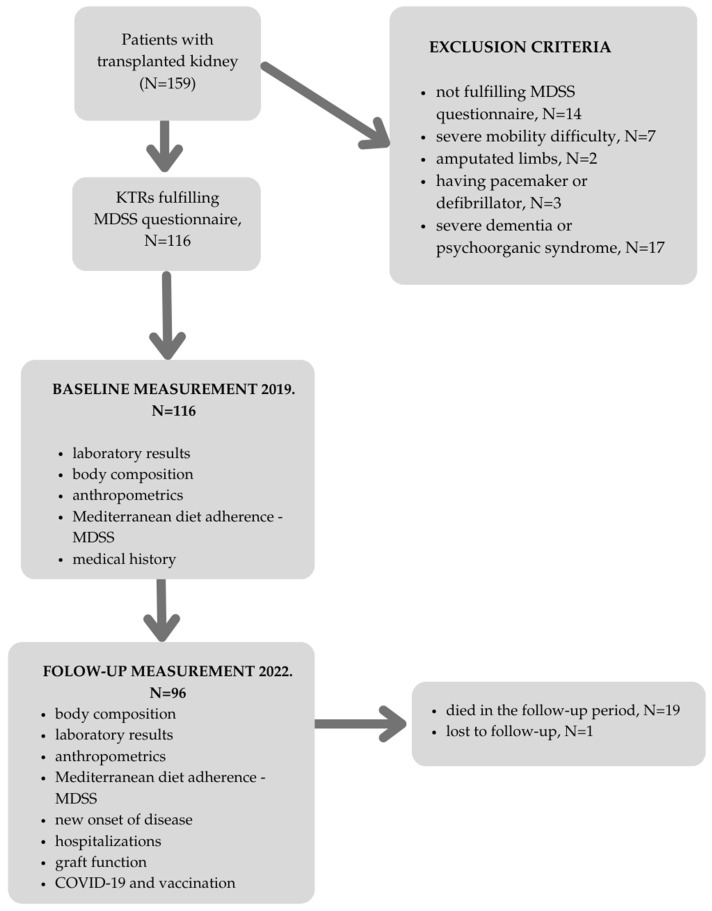
Flow-chart of the study. Abbreviations: N—number, KTR—kidney transplant recipient, MDSS—Mediterranean Diet Serving Score.

All participants were invited to take part in the follow-up study after three years. Nineteen KTRs died during the three years and one participant was lost to follow-up. The study flowchart is shown in Figure 1.

The follow-up examination included bioelectrical impedance body composition analysis, anthropometric measurements, blood pressure measurement, assessment of adherence to the MeDi, and clinical and laboratory evaluations, including outcomes such as death, number of hospitalization, infections, cardiovascular events, graft rejection, malignancies, diabetes, and COVID-19 infection. Vaccination data for COVID-19 were also recorded.

No specific interventions were offered to participants in this study. However, all KTRs at our center received standard post-transplant care. Standard care consists of frequent visits to a specialist physician (with frequency depending on individual time since transplantation, graft function, reporting of complications, and other clinical areas), medication monitoring with measurement of serum concentrations of immunosuppressants, monitoring of blood pressure and weight, monitoring of other comorbidities, and clinically relevant screening for malignancies or infections.

### 2.1. Follow-Up Study Measurements

#### 2.1.1. Body Composition

Each study participant’s body composition was assessed using an MC-780 Multi Frequency Segmental Body Analyzer (Tanita, Tokyo, Japan). The device measures the electrical resistance of various tissues using a steady high frequency current flow and eight electrodes. The procedure is known as bioelectrical impedance analysis. It is used to measure visceral fat, muscle mass (kg), skeletal muscle mass (kg), skeletal muscle mass percentage (%), fat mass (kg), fat free mass (kg), body mass (kg), and PhA (°). Prior to the measurement, all patients were instructed not to consume any food or liquids for at least three hours, to urinate right before the measurement, and not to use alcohol, consume large amounts of food or drink, or engage in extreme exercise for at least one day prior to the measurement [12]. Stadiometers were used to measure height. A flexible, non-stretchable measuring tape was used to measure the subjects’ waist circumference (WC) and mid-upper arm circumference (MUAC) while they were standing, facing forward, and with their shoulders relaxed. Each study participant had their BMI determined.

#### 2.1.2. Mediterranean Diet Adherence

The validated MDSS questionnaire was employed to assess adherence to the MeDi, based on the consumption of various foods and food groups (MeDi components) at specific meal, daily, or weekly intervals. The foods are categorized into fourteen groups, and points are assigned according to the revised Mediterranean food pyramid as follows: three points are awarded for consuming fruits, vegetables, olive oil, and cereals at each meal; two points for daily consumption of dairy products and nuts; and one point for meeting the recommended weekly servings of potatoes (≤3), legumes (≥2), eggs (2–4), fish (≥2), white meat (2), red meat (<2), sweets (≤2), and fermented beverages (1–2 glasses per day). The total MDSS score can reach a maximum of twenty-four (24), with a higher score indicating greater adherence to the MeDi. An optimal cut-off score of ≥13.50 was established to differentiate between adherence and non-adherence to the MeDi [13].

#### 2.1.3. Clinical Outcomes and Laboratory Parameters

Throughout the 3-year follow-up, participants were closely monitored for various clinical outcomes. Data were collected on death outcome, total number of hospitalization, severe infections (requiring hospitalization and/or intravenous antimicrobial or specific antiviral therapy), new-onset cardiovascular disease (first-time diagnosis of conditions like angina pectoris, myocardial infarction, etc.), graft rejection, new-onset malignant disease (excluding non-invasive skin cancers), new-onset diabetes mellitus (diagnosed by fasting blood glucose (FBG) or HbA1c criteria), and COVID-19 infection. Vaccination data for COVID-19 were also recorded.

In terms of laboratory parameters, all participants underwent standard peripheral blood sampling, and they were instructed to provide a 24 h urine sample on the same day as their body composition and blood pressure measurements. The collected data included levels of urea (mmol/L), creatinine (mmol/L), uric acid (mmol/L), serum albumin (g/L), phosphates (mmol/L), C-reactive protein (CRP; mg/L), calcium (mmol/L), FBG (mmol/L), triglycerides (mmol/L), total cholesterol (mmol/L), LDL (mmol/L), number of erythrocytes (×10^12^/L), hemoglobin (g/L), mean cellular volume (MCV), sodium (mmol/L), potassium (mmol/L), phosphate (mmol/L), and estimated glomerular filtration rate (eGFR) calculated using the CKD-EPI equation (mL/min/1.73 m^2^). A complete blood count was performed using a hematology analyzer (Advia 120, Siemens, Erlangen, Germany) at the time of the follow-up.

### 2.2. Statistical Analysis

Categorical variables were presented as frequencies and percentages, while numerical variables were first tested for normality using the Shapiro–Wilk test and presented with means and standard deviations or medians and interquartile ranges (IQRs) when deviated from normality. To test the difference between the two groups (follow-up vs. baseline), the McNemar test was used for categorical variables, whereas the paired *T*-test or Wilcoxon signed rank test was used for numerical variables. Statistical significance was set at *p* < 0.05.

To evaluate the association between the baseline measurements and the risk of future worsening eGFR or lower muscle mass, logistic regression models were performed, whereas to evaluate the effect on survival time (death outcome), a Cox proportional hazards model was used. For the initial variable selection, univariate regression models were applied, adjusted for age and sex. Baseline variables with a *p*-value < 0.1 were retained for further analysis. Participants with missing data on specific dietary variables required for the further analysis were excluded, whereas if any participant had some missing value in any of the required biochemical variables, the MICE (Multiple Imputation by Chained Equation) algorithm for imputation was applied (R package mice v3.17.0) [14]. Subsequently, a stepwise regression analysis with both backward and forward selection was conducted using the selected variables to identify independent predictors. Results of logistic regression models were presented with odds ratios (ORs) and 95% confidence intervals (CIs), where those of the Cox regression model were presented with hazard ratios (HRs) and 95% confidence intervals (Cis). Complete analyses were performed in the free software environment for statistical computing R version 4.3.2 [15], with the following packages for visualizations: ggplot2 v3.5.1 [16] and ggpubr v0.6.0 [17].

## 3. Results

This prospective study included a total of 116 KTRs over the 3-year study period, with one patient being lost to follow-up. Hence, the present analysis is based on 115 KTRs. Out of these, 19 (16.52%) KTRs died during the study period, so further analyses from the follow-up data were made on the sample of 96 KTRs.

The cohort consisted of 51 males (44.3%) and 64 females (55.7%), with a median age of 54 years (IQR 14.25). The demographic and clinical parameters after follow-up are well-summarized in Table 1.

### 3.1. Changes in Mediterranean Diet

Adherence over the 3-Year Follow-Up Period

**Figure 2:**
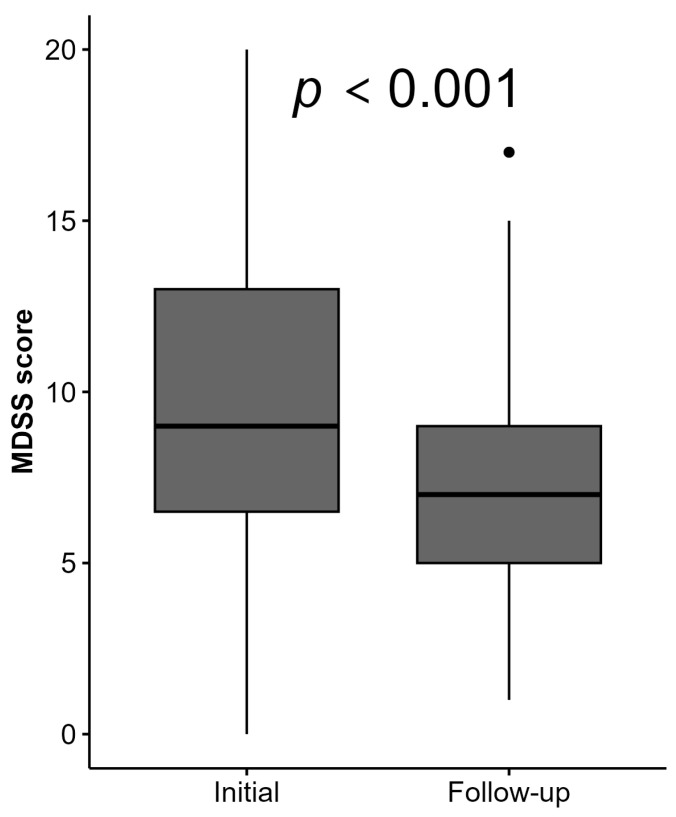
Changes in the MDSS score in a follow-up period in kidney transplant recipients. Abbreviations: MDSS—Mediterranean Diet Serving Score.

**Figure 3:**
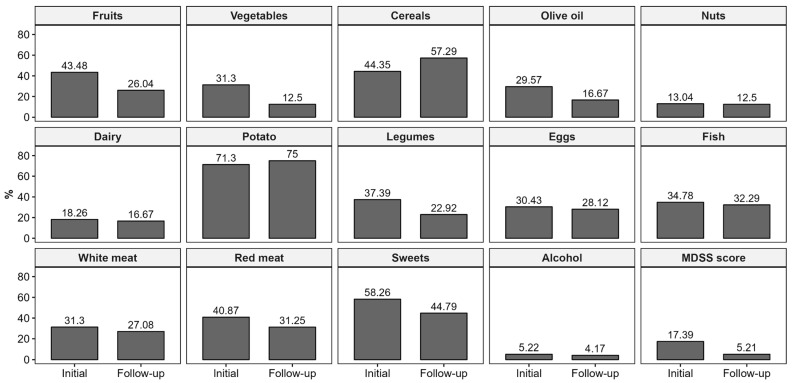
Changes in adherence to different components of the Mediterranean diet in the follow-up period. Abbreviations: MDSS—Mediterranean Diet Serving Score.

After a 3-year follow-up period, the MDSS score significantly decreased in the KTRs, with a median MDSS score of 9 (IQR = 5.75) at baseline versus 7 (IQR = 4) at follow-up, (*p* = 0.001) (Figure 2). Among foods and food groups, there was a deterioration in adherence to MeDi recommendations for fruit, vegetable, legume, and sweet intake (*p* < 0.02), shown in Figure 3 and Supplementary Table S1. Furthermore, the rate for complete MeDi adherence significantly decreased from 15 (18.29%) to 5 (5.95%) (*p* = 0.028), highlighting the challenges of maintaining dietary interventions in this population (Supplementary Table S1).

### 3.2. Changes in Body Composition Parameters

Over the three-year follow-up, significant changes were observed in most of the body composition parameters.

**Figure 4:**
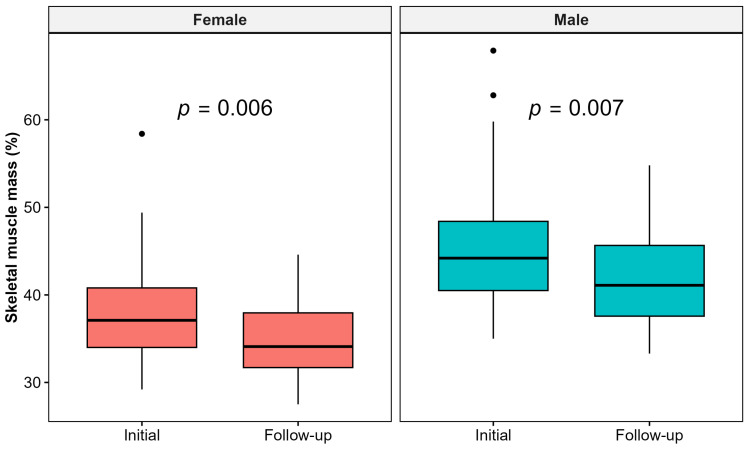
Changes in skeletal muscle mass % in male and female kidney transplant recipients in the follow-up period.

Skeletal muscle mass significantly deteriorated among the cohort (from 33 kg (IQR 12.12)/41.8% (IQR 6.46) to 29 kg (IQR 12.83)/39% (IQR 6.14); *p*-values 0.007 and 0.004, respectively), as presented in Supplementary Table S1. Figure 4 represents the changes in skeletal muscle mass % in male and female KTRs.

Furthermore, patient body water [from 42.75 kg (IQR 13.3)/53.8% (IQR 8.48) to 39.8 kg (IQR 13.85)/51.2% (IQR 8.78), *p*-values 0.029 and 0.016], intracellular water [from 24.65 (IQR 9.02) to 21.4 (IQR 9.65), *p* = 0.003], and extracellular water/intracellular water ratio [from 0.74 (IQR 0.1) to 0.81 (IQR 0.15), *p* < 0.001] significantly increased, whilst the PhA significantly decreased [from 5.2 (IQR 1) to 5 (IQR 1), *p* = 0.007].

Visceral fat and extracellular water practically remained stable, and BMI [from 25.8 (IQR 5.5) to 25.7 (IQR 5.43)] slightly decreased, whilst fat mass [from 19.2 kg (IQR 11.1)/23.4% (IQR 8.59) to 19.3 kg (IQR 12.1)/24.6% (IQR 9.36)] slightly increased, although the margin for statistical significance was not reached for the latter parameters, and this was only a trend.

### 3.3. Changes in Laboratory Parameters

The trends in laboratory parameters, including the hemogram, lipid profile, FBG, mineral levels, and kidney function, are presented in Supplementary Table S1.

**Figure 5:**
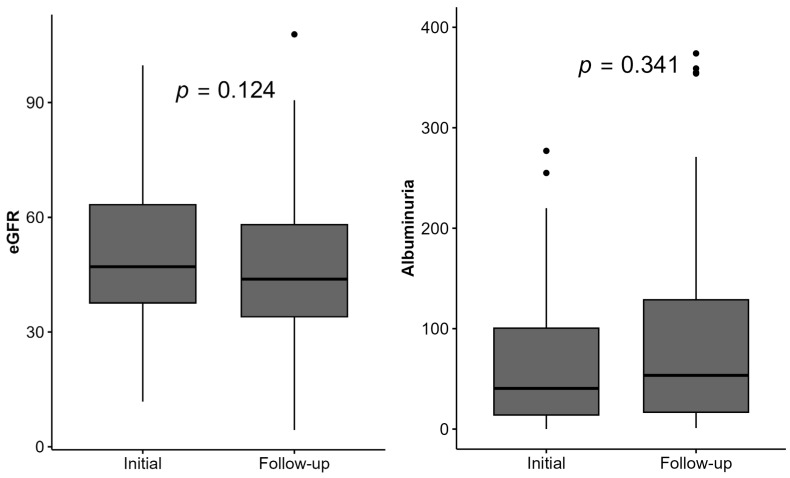
Changes in eGFR and albuminuria level in the follow-up period among all KTRs. Abbreviations: eGFR—estimated glomerular filtration rate using CKD-EPI.

Significant changes in the serum creatinine level, eGFR, and albuminuria during follow-up were not found, as shown in Supplementary Table S1 and Figure 5. Nonetheless, 32 (33%) participants had a higher eGFR, and 56 (58%) had a lower eGFR in the follow-up period.

### 3.4. New-Onset Conditions and Clinical Outcomes

Throughout the 3-year follow-up, participants were closely monitored for various clinical outcomes (Table 1). The observed rate of new-onset conditions were as follows: cardiovascular disease 15 (16.3%), malignant disease 8 (8.7%), diabetes mellitus 1 (1.1%), graft rejection 11 (12.1%), severe clinical infection 37 (40.2%), cerebrovascular disease 2 (2.2%), COVID-19 infection 37 (47.4%), and COVID-19 vaccination 58 (76.3%). A total of 19 participants (16.5%) died during the follow-up period while the median number of total hospitalizations was 1 (IQR = 2).

### 3.5. Associations Between MeDi Adherence, Body Composition, Laboratory Parameters, and Clinical Outcomes

The regression analyses pointed out the predictors for deterioration in the eGFR and skeletal muscle mass, as well as for the death outcome. First, univariate regression models were performed, which were all adjusted for the effects of age and gender. Predictors with a *p*-value < 0.1 were used as input variables for stepwise regression analyses with both backward and forward selection, and the final regression models are shown in Table 2, Table 3 and Table 4.

Several significant predictors of eGFR deterioration were identified, including baseline factors such as SBP, visceral fat, hemoglobin, and years since transplantation. Cereal intake, FBG, cholesterol, LDL, triglycerides, and the presence of diabetes mellitus were shown to be predictive for lower skeletal muscle mass. Lastly, the death outcome among our cohort was associated with the baseline measurement of the PhA, egg intake, cholesterol, and calcium level.

## 4. Discussion

### 4.1. Mediterranean Diet

This prospective study shows a discouraging trend in adherence to the MeDi. Adherence rates dropped to only 5.95% among KTRs in Dalmatia over the three-year period. This alarming decline not only highlights a significant deviation from the MeDi but is also an urgent call to support nutrition for this vulnerable population. The MDSS score dropped from an already low level at baseline to an even lower level, indicating that most participants’ adherence to the Mediterranean diet was suboptimal to begin with and worsened over time. The MeDi is beneficial for KTRs in several aspects. It leads to better health outcomes and helps maintain the graft function. The diet consists of a combination of fruits, vegetables, whole grains, and healthy fats, which promotes cardiovascular health—an important issue for transplant patients who are prone to high cardiovascular risk [1]. Studies show that the rate of CKD and its complications, including hyperphosphatemia and metabolic acidosis, is lower in those who adhere to the MeDi, as fewer uremic toxins and inflammatory factors are synthesized [18].

Diets such as the MeDi, which are rich in plants, also slow the decline in renal allograft function over time, perhaps due to their nutrient composition [2]. Moreover, this polyphenol-rich diet has antioxidant and anti-inflammatory properties that could be useful in treating post-transplant complications and improving graft function [19]. In summary, a MeDi is associated with improved metabolic health and may have a positive impact on the psychological well-being of KTRs [3]. KTRs in our study have reduced their adherence to the MeDi. Obstacles to following the MeDi can be a lack of access to healthy foods, high expenses, low income, and demographic issues including gender, age, and educational attainment. Cognitive difficulties, including ignorance of the advantages and ingredients of the diet, as well as sensory and cultural preferences might be influential. Access to food outlets, the diet’s stated health and environmental advantages, affordability, and motivating elements like self-efficacy and adaptability are all considered facilitators [20].

MeDi adherence was reduced especially for the recommended intake of fruits, vegetables, olive oil, legumes, and sweets. These changes could also be due to the increase in food price.

Worsening dietary patterns could have been a product of the COVID-19 pandemic.

Di Renzo et al. and Skotnicka et al. demonstrated notable changes in food patterns and lifestyle choices made by various European communities during the COVID-19 pandemic. Younger Italians reported a higher adherence to the MeDi and a sense of weight gain, according to Di Renzo’s research, while Skotnicka’s survey found that Poland, Austria, and the UK had higher dairy and frozen food consumption, lower levels of physical activity, and higher alcohol consumption [21,22].

The online study from Croatia, inversely to our results, shows increased cooking frequency, and an increase in vegetable and legume, as well as fish and seafood consumption during the confinement [23].

Due to the mentioned pandemic, KTRs who are immunosuppressed might have prioritized hygiene, wearing masks, and avoiding big groups. On the topic of their awareness of COVID-19 speaks the fact that more than three-quarters of them were vaccinated.

### 4.2. Body Composition Parameters

Regarding body composition, the results of our study show a significant deterioration of muscle mass in KTRs at the 3-year follow-up. We also found lower levels of PhA in the follow-up period.

Reduced muscle mass over an extended period, like a 3-year follow-up, is a significant issue for KTRs, as several studies clearly show a correlation between continued muscle loss and worsening outcomes. Dienemann et al. [24] found that, although there are early improvements in muscle mass following transplantation, they are often surpassed by fat growth, leading to permanent relative sarcopenia. This dynamic illustrates how KTRs’ body composition deteriorates with time, increasing the risk of cardiovascular disease, decreasing muscular function, and lowering physical performance. It could also be caused by the COVID-19 pandemic and isolation for a long period of time, especially for this vulnerable immunocompromised population.

Interventions to prevent the loss of muscle mass by using specific dietary and activity plans have also shown promise. According to Kosoku et al. [25], maintaining muscle mass in KTRs over time requires a sufficient protein intake. More specifically, post-transplant improvements in skeletal muscle mass were linked to sustaining a daily protein intake of at least 0.72 g/kg ideal body weight. Insufficient protein intake made KTRs in their research more likely to lose muscle mass, which affected their ability to recover functionally and their physical strength.

Exercise has also been shown to be essential for reducing muscle loss. According to Lima et al. [26], combined aerobic and resistance training greatly enhanced muscle mass and strength in KTRs while lowering body fat. This suggests that organized physical activity may be able to mitigate the susceptibility to lose muscle after transplantation. Longer term, these programs could help with muscle preservation and functional ability, especially for patients who are more likely to lead sedentary lives after receiving a transplant due to health-related constraints.

A prospective study on 31 KTRs by Fukhura et al., who studied changes in body composition parameters assessed with computed tomography before KT and at 2, 4, and 6 years post-transplantation, found a 1.30-fold increase from baseline 2 years after transplantation in subcutaneous visceral adipose tissue and a 1.47-fold increase in visceral adipose tissue 2 years after KT, with a further 1.75-fold increase 6 years after KT. In contrast, muscle quality, represented by skeletal muscle radiodensity, decreased, with a 0.86-fold decrease from baseline after 2 years [27]. In contrast to them, our results do not show a significant increase in fat mass or visceral fat assessed by BIA in a 3-year follow-up, but we did find associations between worsening eGFR and visceral fat level. When considering these results, the different methods used in the two studies need to be considered.

PhA is a useful indicator of muscle integrity and cellular health in KTRs; lower PhA values correspond to decreased muscle mass and strength. Research indicates a negative correlation between a lower PhA in KTRs and a higher risk of sarcopenia, a decline in functional performance, and a worse overall quality of life over time [28,29].

Another important observation from our study is the finding that a lower PhA in the baseline measurement was associated with a greater risk of the death outcome, as the results of the Cox regression analysis show.

This result adds to the knowledge on the importance of the PhA as a prognostic marker in this population.

Like our results, among 158 KTRs from a study by Kaya et al., the PhA was substantially associated with death throughout an 8-year follow-up period, with a cutoff value of ≤5.85. Furthermore, a PhA value less than 5.85 suggested a 5.33-fold increased risk of death [30].

### 4.3. Clinical and Laboratory Parameters

Regarding the laboratory parameters, there was no significant change in the eGFR and albuminuria in the follow-up period.

A multicentric study on more than 4000 KTRs in Spain showed a very slow decline in the rate of graft function (−1.12 ± mL/min per 1.73 m^2^ per year) [31].

Furthermore, the levels of total cholesterol and LDL cholesterol decreased. The reason for this can be the greater prescription and adherence to lipid-lowering therapy. The baseline cholesterol level, found as a predictor of the death outcome in our study, is in line with previous knowledge [32].

Furthermore, the most frequent new-onset disease in the 3-year follow-up was a serious clinical infection, which affected 40.22% of KTRs. These results are in line with a follow-up study on more than 6000 KTRs in Canada, which found infectious diseases and endocrine disorders as the two most common causes of hospitalizations [10].

### 4.4. Strengths and Limitations

The primary advantage of this research is its longitudinal design, which enables the monitoring and assessment of changes in dietary adherence and body composition within a 3-year period among a specific cohort of KTRs. In addition to this, usage of a number of in-depth validated MeDi adherence and body composition measurement instruments further enhance the quality of our results. Nonetheless, several limitations should be recognized. The observational design of the study prevents us from drawing any causal conclusions between MeDi adherence and the outcomes observed. Furthermore, some external variables, like the COVID-19 pandemic, may have influenced dietary patterns and lifestyle behaviors, potentially confounding the observed results.

To review, when discussing clinical implications, this study offers a valuable insight in the worsening of dietary patterns and body composition in the follow-up period, implying an aggravated need for action. Also, it provides a direction for future research on the assessment of clinical parameters of KTRs.

## 5. Conclusions

Maintaining adherence to the MeDi diet presents noteworthy challenges in the case of KTRs, with adherence rates declining sharply over time. The latter deterioration appears to be linked with a substantial decrease in skeletal muscle mass and PhA, which are important variables of muscle health and cellular integrity. The results obtained highlight the need for targeted interventions to support dietary and physical well-being in KTRs, and potentially improve clinical outcomes in this vulnerable population.

## Figures and Tables

**Table 1:** General characteristics of the follow-up.

Variable	All (N = 115)
Alive, N (%)	96 (83.5)
Death outcome, N (%)	19 (16.5)
Male sex, N (%)	51 (44.4)
Age (years), median (IQR)	63 (16)
pSBP (mmHg), median (IQR)	140.0 (30.0)
pDBP (mmHg), mean (SD)	82.9 (15.2)
Waist circumference (cm), median (IQR)	99.0 (15.5)
Upper arm circumference (cm), median (IQR)	31.0 (4.5)
Height (cm), mean (SD)	173.8 (10.2)
Weight (kg), median (IQR)	76.7 (21.2)
BMI (kg/m^2^), mean (SD)	25.9 (4.0)
Body Composition	
Fat mass (kg), median (IQR)	19.3 (12.1)
Fat mass (%), mean (SD)	24.6 (9.4)
Fat free mass (kg), median (IQR)	59.5 (19.2)
Visceral fat level, median (IQR)	9.0 (5.0)
Muscle mass (kg), median (IQR)	55.8 (18.3)
Skeletal muscle mass (kg), mean (SD)	30.1 (8.0)
Skeletal muscle mass (%), mean (SD)	39.0 (6.1)
Phase angle (°), median (IQR)	5.0 (1.0)
Laboratory Parameters	
E (×10^12^/L), mean (SD)	4.7 (0.7)
Hemoglobin (g/L), mean (SD)	130.8 (18.2)
MCV (fL), median (IQR)	87.5 (6.4)
Urea (mmol/L), median (IQR)	9.9 (6.6)
Creatinine (µmol/L), median (IQR)	130.0 (58.3)
eGFR using CKD-EPI (mL/min/1.73 m^2^), mean (SD)	43.85 (24.1)
FBG (mmol/L), median (IQR)	5.3 (1.6)
Albumin (g/L), mean (SD)	42.3 (3.2)
CRP (mmol/L), median (IQR)	2.2 (3.9)
Cholesterol (mmol/L), mean (SD)	5.2 (1.0)
LDL (mmol/L), mean (SD)	2.9 (0.9)
Triglycerides (mmol/L), median (IQR)	1.8 (1.2)
Sodium (mmol/L), median (IQR)	141.0 (5.0)
Potassium (mmol/L), median (IQR)	4.2 (0.7)
Calcium (mmol/L), median (IQR)	2.4 (0.2)
Phosphate (mmol/L), median (IQR)	1.0 (0.3)
Uric acid (mmol/L), mean (SD)	384.4 (88.1)
Albuminuria (mg/dU), median (IQR)	65.0 (126.8)
New-onset cardiovascular disease, N (%)	15 (16.3)
New-onset malignant disease, N (%)	8 (8.7)
New-onset diabetes, N (%)	1 (1.1)
New-onset disease—graft rejection, N (%)	11 (12.1)
New-onset severe clinical infection, N (%)	37 (40.2)
New-onset cerebrovascular disease, N (%)	2 (2.17.0)
Number of hospitalizations in the follow-up period, median (IQR)	1 (2)
Had COVID-19, N (%)	37 (47.4)
Vaccinated for COVID-19, N (%)	58 (76.3)
MDSS score, median (IQR)	7 (4)
Adherence to the MeDi, N (%)	5 (6.0)

Abbreviations: pSBP—peripheral systolic blood pressure, pDBP—peripheral diastolic blood pressure, BMI—body mass index, E—erythrocyte count, MCV—Mean Corpuscular Volume, eGFR using CKD-EPI—estimated glomerular filtration rate calculated using the Chronic Kidney Disease Epidemiology Collaboration equation, FBG—fasting blood glucose, CRP—C-reactive protein, LDL—low-density lipoprotein cholesterol, COVID-19—coronavirus disease 19, MDSS—Mediterranean Diet Serving Score, MeDi—Mediterranean diet.

**Table 2:** Predictors of death outcome—results from Cox regression.

	Beta	OR (95% CI)	*p*
Egg intake	−1.06	0.35 (0.14–0.87)	0.023
Phase angle (°)	−2.68	0.07 (0.01–0.43)	0.004
Cholesterol (mmol/L)	0.95	2.60 (1.40–4.70)	0.001
Calcium (mmol/L)	−7.21	0.00 (0.00–1.50)	0.063

Abbreviations: OR—odds ratio, CI—confidence interval.

**Table 3:** Predictors of worsening eGFR in the follow-up period—logistic regression.

	Beta	OR (95% CI)	*p*
SBP (mmHg)	0.04	1.04 (1.01–1.08)	0.021
Visceral fat	−0.18	0.84 (0.71–0.98)	0.032
Hemoglobin (g/L)	0.06	1.06 (1.02–1.10)	0.008
Time since transplantation (years)	0.16	1.17 (1.01–1.35)	0.034

Abbreviations: OR—odds ratio, CI—confidence interval, SBP—systolic blood pressure.

**Table 4:** Predictors of lower muscle mass in the follow-up period—logistic regression.

	Beta	OR (95% CI)	*p*
Cereal intake	1.24	3.46 (0.93–12.88)	0.065
FBG (mmol/L)	−0.45	0.64 (0.33–1.22)	0.171
Cholesterol (mmol/L)	2.71	15.03 (1.23–183.95)	0.034
LDL (mmol/L)	−3.52	0.03 (0.00–0.55)	0.018
Triglycerides (mmol/L)	−0.96	0.38 (0.15–0.96)	0.041
Diabetes mellitus	−1.42	0.24 (0.05–1.18)	0.080

Abbreviations: OR—odds ratio, CI—confidence interval, FBG—fasting blood glucose, LDL—low-density lipoprotein cholesterol.

## Data Availability

Data are available upon reasonable request to the corresponding author’s e-mail.

## Supplementary Materials

The following supporting information can be downloaded at: https://www.mdpi.com/article/10.3390/healthcare13070840/s1, Table S1: Descriptive statistics and regression analysis.
